# Epidemiology of Knee Injuries in Baseball Players from the State of São Paulo

**DOI:** 10.1055/s-0044-1785202

**Published:** 2024-04-10

**Authors:** Victor Kenzo Arashiro, Karin Coca Aguilar, Tian Xu, Nayra Deise dos Anjos Rabelo, Alfredo dos Santos Netto, Ricardo de Paula Leite Cury

**Affiliations:** 1 Departamento de Ortopedia e Traumatologia, Faculdade de Ciências Médicas Santa Casa de Misericórdia de São Paulo (FCMSCSP), São Paulo, SP, Brasil; 2 Núcleo de Apoio à Pesquisa em Análise do Movimento (NAPAM), Universidade Nove de Julho (Uninove), São Paulo, SP, Brasil

**Keywords:** baseball, knee, knee injuries, tibial meniscus injuries, tendinopathy

## Abstract

**Objective** This study aimed to identify the main knee complaints and injuries associated with baseball and their prevalence in the state of São Paulo, Brazil.

**Methods** This epidemiological study analyzed data from an online questionnaire sent to baseball athletes from the state of São Paulo, Brazil, from 2019 to 2022.

**Results** Ninety-eight athletes participated in the study. Their average age was 24.3 years, and 85.72% of the subjects were men. The most prevalent ethnicities were yellow (50%) and white (42.86%). Most athletes had incomplete or complete higher education (75.5%). Most (88.77%) have been training for over 1 year, and 40.82% played in more than 1 position. More than half also practiced another sport. Most (66.32%) athletes present knee complaints or symptoms, and 37.75% had suffered a knee injury playing baseball, with several mechanisms (contact with the ground, contact with another player, or no contact). More than half (59.45%) of the athletes required time away from baseball due to complaints, symptoms, or injuries.

**Conclusion** Among the athletes interviewed, 66.32% had a knee complaint, and 37.75% had already had a knee injury, especially meniscal and ligament injuries. The injury rate was highest in the first year of practice.

## Introduction

Musculoskeletal injuries are common in sports, accounting for approximately 80% of sports injuries. 1 Joint injuries, especially in the knee, have increased significantly due to the growth in the number of people who practice physical activity both professionally and recreationally. Furthermore, these practices start at an increasingly younger age group, with a demand for training and increasingly higher levels of competitiveness, 2 3 sometimes lacking proper technical supervision.

Studies on baseball injuries focus on the upper limbs, which correspond to approximately 45% of injuries occurring during training and games. Eighty percent of articles published based on Major League Baseball are related to the upper limbs, while 6.6% refer to the lower limbs. 4 However, lower limb injuries are also present in this sport, corresponding to around 33% of the total injuries in this modality. 5 6 7 Despite this, the literature on this topic is limited.

Given the scarcity of literature on knee injuries in baseball and the absence of precise national epidemiological data that would allow us to understand their panorama, this work aimed to identify the main complaints and injuries associated with baseball and their prevalence in our environment, starting a discussion about the need to implement preventative measures for safe practice.

## Methods

From May 2019 to May 2022, after approval by the Ethics and Research Committee (CAAE: 17721819.0.0000.5479), researchers from the Knee Group of the Department of Orthopedics and Traumatology of the same hospital asked several baseball clubs in the state of São Paulo and university teams to distribute an online questionnaire among their athletes. The Google Forms questionnaire included a digital version of the informed consent form and specific questions for demographic, epidemiological identification, and knee injuries. The questionnaire was developed and evaluated internally by group members over three rounds of review. The final version was sent directly to athletes to collect data related to baseball practice.

Amateur athletes from Liga Esportiva das Atléticas de Medicina do Estado de São Paulo (LEAMESP) and the Anhanguera, Atibaia, Gecebs, Nippon Blue Jays, São Paulo Gigante, Los Tomateros, Underdogs, and UniSant'Anna clubs received an invitation to voluntarily self-complete the questionnaire ( Supplementary Material, Annex 1 ). The questionnaire was sent by WhatsApp to the representative of each institution, reaching a total of 195 athletes.

The inclusion criteria were amateur baseball athletes from the state of São Paulo, regardless of age, gender, or ethnicity, who practice the sport at least once a week at different levels of performance, and affiliated with the clubs above, and who answered the questionnaire and consent form completely and correctly. The previously defined exclusion criteria were athletes with musculoskeletal injuries resulting from another sport, those who did not answer the questionnaire as requested, and para-athletes, that is, those with physical, visual, or intellectual impairment.

### Questionnaire Description

The questionnaire prepared by the authors consisted of 23 questions: 4 on demographic data, 6 on sports practice, and 13 on specific symptoms, diagnoses, and treatments for knee injuries. This questionnaire aimed to identify epidemiological issues involving this population.

The demographic data section requested information on gender, age, ethnicity, and education. The section on baseball sports practice collected data on time spent practicing the sport (more than or less than a year), time dedicated to training in weekly hours, and the playing position—pitcher, catcher, infielder (first base, second base, third base, and shortstop) and outfielder (left fielder, center fielder, and right fielder). Data collection also included information on the practice of other sports, training time in hours per week, and practice time, either more or less than a year.

The questionnaire also asked about specific knee symptoms, diagnoses, and treatments, such as knee-related clinical complaints (including pain and topography, episodes of instability, edema, locking, and crepitus), specific injury diagnoses throughout baseball practice, a description of these diagnoses if more than one, injury mechanisms (non-contact, contact with the ball, ground, object, another player, and others), time away from baseball due to clinical complaint or injury, length of absence in months, performance of conservative treatments and their nature, and surgical treatments and their descriptions.

### Data Analysis

As for demographic data, age was shown as mean and standard deviation. Gender, ethnicity, education, sports practice, specific knee symptoms, diagnoses, and treatments were displayed in absolute and relative numbers.

The remaining data were tabulated and displayed as previously explained. We analyzed age, time of baseball practice in years, weekly training hours, playing position, and practice of another sport to highlight potential associations between complaints or injuries and some study variables.

For this secondary analysis, we stratified participants into different subgroups and observed the distribution of complaints and injuries in each one. These subgroups referred to median age and baseball practice time for less than a year or equal or more than a year. This division was based on studies showing differences in injury rates in athletes stratified this way. 8 9 Weekly training hours comprised five categories. We also analyzed the participation in one or more baseball positions. Regarding other sports, participants were subdivided into groups only playing baseball and practicing additional modalities.

## Results

Ninety-eight of 195 athletes completed the questionnaire voluntarily, corresponding to a response rate of 50.25%. The mean age of participants was 24.3 ± 7.1 years. The sample included 84 (85.72%) men and 14 (14.28%) women. No athlete was excluded after completing the questionnaire. There were 4 distinct ethnicities, that is, 49 (50%) yellow, 42 (42.86%) white, 6 (6.12%) brown, and 1 (1.02%) black. Among the athletes, 75.5% declared that they had incomplete or complete higher education ( Table 1 ).

**Table 1:** Demographic data in mean, standard deviation and median or absolute and relative numbers of participants

**Age**	**Mean (SD)**	**Median**			
	24.33 (±7.1)	23			
Gender	Male	Female			
	84 (85.72%)	14 (14.28%)			
Ethnicities	Yellow	White	Brown	Black	
	49 (50%)	42 (42.86%)	6 (6.12%)	1 (1.02%)	
Education	Incomplete elementary education	Incomplete high school education	Complete high school education	Incomplete college education	Complete college education
	5 (5.1%)	4 (4.08%)	15 (15.4%)	52 (53.06%)	22 (22.44%)

Abbreviations: SD, Standard deviation; %, relative number in percentage.

Regarding the specific practice of baseball, 87 (88.77%) athletes trained for more than a year, and 24 (24.48%) trained more than 10 hours a week. Forty (40.82%) subjects played in at least two positions; most athletes played as infielders or outfielders (51.02% and 47.95%, respectively), and 50 (51.02%) athletes practiced other sports ( Table 2 ).

**Table 2:** Data regarding baseball practice in absolute and relative numbers

**Practicing time (N = 98)**	**Less than a year**	**Over a year**			
	11 (11.23%)	87 (88.77%)			
Training time (hours per week) (N = 98)	Up to 5 hours	5–10 hours	10–15 hours	15–20 hours	Over 20 hours
	36 (36.73%)	38 (38.77%)	17 (17.34%)	6 (6.12%)	1 (1.02%)
Positions*	Pitcher	Catcher	Infielder	Outfielder	
	30 (30.61%)	18 (18.36%)	50 (51.02%)	47 (47.95%)	
Field positioning (N = 98)	Single	Multiple			
	58 (59.18%)	40 (40.82%)			
Other sports (N = 98)	Yes	No			
	50 (51.02%)	48 (48.97%)			
Training time (hours per week) in other sport (N = 50)	Up to 5 hours	5–10 hours	10–15 hours	15–20 hours	Over 20 hours
	21 (42%)	18 (36%)	7 (14%)	3 (6%)	1 (2%)
Practicing time in other sport (N = 50)	Less than a year	Over a year			
	4 (8%)	46 (92%)			

Abbreviations: SD, Standard deviation; %, relative number in percentage. *participants may play in more than one position.

Sixty-five athletes (66.32%) complained of knee symptoms. Thirty-seven (37.75%) had already been diagnosed with a baseball-associated knee injury, including 14 (37.83%) with ligament injuries, 14 (37.83%) with several meniscal injuries, and 10 (27.02%) with tendinopathies. It is worth mentioning that 16 (43.24%) athletes already diagnosed declared the occurrence of more than one injury. Regarding trauma mechanisms, 10 (45.45%) athletes suffered an injury due to contact with the ground, 8 (36.36%) reported no contact, and 4 (18.18%) had contact with another player. Twenty-two (59.45%) reported the need to take time off from sports due to clinical complaints or injuries, of which 5 (22.72%) underwent surgical treatment ( Table 3 ).

**Table 3:** Data regarding specific knee symptoms, diagnoses, and treatments in absolute and relative numbers

**Symptoms**	**None**	**Anterior pain**	**Posterior pain**	**Lateral pain**	**Edema**	**Instability**	**Locking**	**Crepitation**
	42 (33.9%)	49 (39.5%)	11 (8.9%)	36 (29%)	9 (7.3%)	15 (12,10%)	3 (2.4%)	44 (35.5%)
**Diagnosed injury**	Yes	No						
	37 (37.75%)	61 (62.24%)						
**Injuries (N = 37)**	Tendinopathy	ACL rupture	PCL rupture	LCL rupture	MCL rupture	Multiligamentous lesions	Meniscus	Osteochondritis dissecans
	10 (27.02%)	10 (27.02%)	3 (8.1%)	2 (5.4%)	1 (2.7%)	2 (5.4%)	14 (37.83%)	0 (0%)
	ITBS	Patellar chondropathy	Patellar dislocation	Osgood Schlatter	Baker cyst			
	0 (0%)	10 (27.02%)	7 (18.91%)	3 (8.1%)	1 (2.7%)			
**Mechanisms of knee injury (N = 22)**	Contact with the ball	Contact with the ground	Contact with an object	Contact with another player	No contact			
	0 (0%)	10 (45.45%)	0 (0%)	4 (18.18%)	8 (36.36%)			
**Time away from baseball resulting from knee injury (N = 37)**	Yes	No						
	22 (59.45%)	15 (40.54%)						
**Time away from baseball (N = 22)**	Up to 1 month	1–3 months	3–6 months	Over 6 months				
	7 (31.81%)	8 (36.36%)	4 (18.18%)	3 (13.63%)				
**Non-surgical treatment due to knee injury (N = 37)**	Yes	No						
	21 (56.75%)	16 (43.25%)						
**Surgical treatment due to knee injury (N = 37)**	Yes	No						
	5 (13.51%)	32 (86.49%)						

Abbreviations: ACL, anterior cruciate ligament; LCL, lateral collateral ligament; MCL, medial collateral ligament; PCL, posterior cruciate ligament; N, number of participants in the group; %, relative number in percentage; ITBS, iliotibial band syndrome.

For the secondary analysis, we initially subdivided the athletes into 2 groups based on their median age, 23 years old ( Table 1 ). Among athletes older than 23, 71.11% had clinical complaints regarding their knees, and 35.55% had already been diagnosed with a knee injury.

We noted that 72.72% (eight out of 11) of athletes who have played baseball for less than a year had clinical knee complaints, and 45.45% (five out of 11) had already been diagnosed with a knee injury. Among athletes practicing for more than a year, 65.51% reported complaints (57 of 87), and 36.78% had injuries (32 of 87) ( Table 4 ).

**Table 4:** Presence of symptoms and diagnosed injuries concerning practice time

	All (N = 98)	Over a year (N = 87)	Less than a year (N = 11)
Symptoms	65 (66.32%)	57 (65.51%)	8 (72.72%)
Diagnosed injuries	37 (37.75%)	32 (36.78%)	5 (45.45%)

Abbreviations: SD, standard deviation; %, relative number in percentage.

Understanding the limitation of the subject distribution by subgroups with complaints and injuries per weekly training hours, we highlight that 66.66% of the 36 athletes who trained up to 5 hours per week had complaints, including 54.16% with diagnosed injuries ( Table 5 ). Furthermore, of the 40 athletes training in more than one position, 72.5%, and 42.5% reported experiencing knee symptoms or injuries, respectively; of the 50 who played other sports, 66% had complaints, and 34% reported injuries. Among the 48 subjects who only played baseball, 66.66% had symptoms, and 39.58% presented diagnosed injuries.

**Table 5:** Relationship between baseball training time (in hours per week) and the presence of symptoms and diagnosed injuries in absolute and relative numbers

Hours per week	Symptoms	Injuries
Up to 5 hours (N = 36)	24 (66.66%)	13 (54.16%)
5–10 hours (N = 38)	25 (65.78%)	12 (31.57%)
10–15 hours (N = 17)	12 (70.58%)	6 (35.29%)
15–20 hours (N = 6)	3 (50%)	3 (50%)
Over 20 hours (N = 1)	1 (100%)	1 (100%)

Abbreviations: SD, Standard deviation; %, relative number in percentage.

## Discussion

This study aimed to show epidemiological data on knee symptoms and musculoskeletal injuries in amateur baseball athletes in the state of São Paulo, Brazil, to assist their health care and contribute with information for creating preventive measures and regulations for baseball practice. As far as we know, this is the first study to evaluate the epidemiology of knee injuries in baseball athletes in a Brazilian context.

According to Law 9,615 of 1998 of the Brazilian Federal Government providing general rules for sports practice, professional athletes are those receiving remuneration defined in a formal employment contract. Therefore, all athletes in this study qualify as non-professionals (amateurs).

In the main baseball leagues from the United States leagues (Major League Baseball and Minor League Baseball), shoulder and knee injuries account for 14.7% and 6.5% of all lesions. 6 These athletes, despite predominantly using their upper limbs for the throwing movement, also perform intense effort on the lower limbs for straight-line running, sudden direction changes when running, jumping, and, depending on the playing position, remaining crouched for long periods, maintaining or performing knee flexion movements repeatedly. 10 Even throwing effectiveness closely relates to the energy transfer from the lower limbs to the trunk. 11 Thus, the knee is susceptible to injuries in baseball. However, these injuries remain underdiagnosed as there is little research on the topic.

A Japanese study 12 analyzed young baseball athletes reporting pain in the shoulder, elbow, or both. The authors observed that 51.9% of them also complained of knee pain. In our study, 66.32% of all athletes had knee complaints, regardless of whether they reported upper limb symptoms, and 37.5% of participants had a diagnosed injury. A study performed on the database of injuries diagnosed in baseball professional players from North American leagues reported a knee injury rate of only 6.5%, 6 showing the heterogeneity in injury rates in different contexts.

The literature reports that most knee injuries in baseball athletes are contusions (30.5%), 6 ligament injuries (17.5%), 6 tendinopathies (16.9%), 6 and meniscal injuries (9.2–33%). 1 6 The incidence of these conditions varies depending on the study location. Patellofemoral dislocation and subluxation (1.4%) 6 have a lower prevalence. In our study, the main injuries included ligament (37.83%) and meniscal (37.83%) injuries, in addition to tendinopathies (27.02%).

We believe that the amateur nature of baseball practice at the regional level may account for the higher incidence of knee complaints and injuries in our study compared with the international literature, which mainly evaluates professional athletes. 4 6 This discrepancy makes us question the need for more studies focusing on knee injuries in amateur baseball athletes.

Regarding practice time, we found that athletes who trained for less than a year have a higher rate of diagnosed knee injuries. This phenomenon also occurs in other sports, such as road running, in which studies performed since the 1980s concluded that less experienced athletes tend to suffer a higher number of lower limb injuries. 13 14 More recent studies showed that this incidence can be up to twice as high in beginner athletes, that is, during the first year of practice. 8 9 Despite the sample heterogeneity, we can infer that athletes with less practice time have a higher injury rate, even though there are discrepancies in injury characterization, classification in terms of performance level, and difficulty in evaluating the moment of occurrence. Therefore, further studies are required in this area to promote differentiated care for athletes with less practice time.

Regarding the trauma mechanism of the diagnosed injuries, Dahm et al. 6 pointed out that the most significant ones were non-contact (43.9%) and contact with the ground (18.8%). Our study reported 36.36% and 45.45% of mechanisms with no contact and contact with the ground, respectively. Dick et al. 5 stated that 42% of baseball injuries occurred with no contact, and 45% occurred with contact. The main injuries diagnosed in our study were knee ligament and meniscal injuries, often associated with torsional trauma, corresponding to the non-contact trauma mechanism, and corroborating the described scenario.

One injury described in baseball athletes is called catcher's knee. 15 It is little known and, therefore, may be underdiagnosed. The catcher's knee is an osteochondritis dissecans of the posterior region of the femoral condyle resulting from persistent, repetitive joint hyperflexion, a typical movement mechanism for athletes in this position. This study specifically asked about this diagnosis in the knee symptoms and injuries section. The catchers did not present a specific pattern of diagnosis of injuries or complaints, and we did not have any cases of catcher's knee in our sample.

The participants in this study presented high rates of symptoms, injuries, or both diagnosed in the knees, consistent with other studies evaluating the upper limbs of high-performance athletes. 4 5 6 Today, as far as the authors know, Brazil has no guidelines referring to baseball or the use of equipment to prevent knee injuries. This lack may contribute to the difference in data observed in amateur and professional athletes or to the lack of studies focused on the epidemiology of these injuries.

Our study had some limitations, such as the small and heterogeneous sample despite the wide distribution of the questionnaire across leagues and the difficulty in interpreting and associating the presence of complaints and injuries with variables such as playing position, since the same participant may act in different positions. Although this study provided insights about knee injuries and amateur baseball in Brazil, we believe further studies are required with sample calculation and probably a larger sample size to obtain more precise relationships and analyses.

## Conclusion

Among the athletes interviewed, 66.32% reported knee complaints, and 37.75% had a previous knee injury diagnosed. The most prevalent conditions were meniscal and ligament injuries. The injury rate was higher in the first year of baseball practice.

## Supplementary Material

Material Suplementar, Anexo 1 Material Suplementar, Anexo 1

Supplementary Material, Annex 1 Supplementary Material, Annex 1
