# Supplementary material for: Epidemiology of Knee Injuries in Baseball Players from the State of São Paulo
Source: Rev Bras Ortop (Sao Paulo). 2024 Apr 10;59(2):e199–205. doi: 10.1055/s-0044-1785202 (PMC11006523; doi:10.1055/s-0044-1785202)
Supplement: Supplementary file 1 — Material Suplementar, Anexo 1 [file 10-1055-s-0044-1785202-s2300208pt.pdf]

ANEXO 1

QUESTIONÁRIO - Dor no joelho em atletas de Beisebol

Dados Demográficos

1. Idade:
2. Sexo: ☐ Masculino ☐ Feminino
3. Raça / Cor / Etnia: ☐ Amarela ☐ Branca ☐ Parda ☐ Preta ☐ Indígena ☐ Outro:
4. Escolaridade:
   - ☐ Ensino Fundamental Incompleto
   - ☐ Ensino Fundamental Completo
   - ☐ Ensino Médio Incompleto
   - ☐ Ensino Médio Completo
   - ☐ Ensino Superior Incompleto
   - ☐ Ensino Superior Completo

Atividade

5. Em que posição você joga?
   - ☐ Pitcher
   - ☐ Catcher
   - ☐ Primeira Base
   - ☐ Segunda Base
   - ☐ Shortstop
   - ☐ Terceira Base
   - ☐ Left Fielder
   - ☐ Center Fielder
   - ☐ Right Fielder

6. Quantas horas por semana você treina beisebol?
   - ☐ Até 5h
   - ☐ Entre 5 e 10h
   - ☐ Entre 10 e 15h
   - ☐ Entre 15 e 20h
   - ☐ Mais que 20h

7. Há quanto tempo você treina beisebol?
   - ☐ Menos de 12 meses
   - ☐ Mais de 12 meses

8. Você pratica outros esportes? Quais? ☐ Não ☐ Sim: ______________________

9. Quanto tempo você pratica outros esportes fora o beisebol por semana?
   - ☐ Até 5h
   - ☐ Entre 5 e 10h
   - ☐ Entre 10 e 15h
   - ☐ Entre 15 e 20h
   - ☐ Mais que 20h

10. Há quanto tempo você pratica esses outros esportes?
   - ☐ Menos de 12 meses
   - ☐ Mais de 12 meses

 © 2024. The Author(s).

Sintomas e Diagnósticos

11. Você tem alguma queixa ou sintoma envolvendo o joelho?

☐ Nenhum
☐ Dor na parte da frente do joelho
☐ Dor na parte de trás do joelho
☐ Dor nas laterais do joelho
☐ Edema (inchaço)
☐ Falseio
☐ Travamento
☐ Crepitação (estalo)

12. Quantas vezes você já lesionou (por trauma) o joelho? ________________

13. Qual foi o mecanismo de lesão do joelho?

	Sem contato	Contato com o solo	Contato com a bola	Contato com outro jogador	Contato com objeto	Outro
**Lesão 1**	☐	☐	☐	☐	☐	☐
**Lesão 2**	☐	☐	☐	☐	☐	☐
**Lesão 3**	☐	☐	☐	☐	☐	☐
**Lesão 4**	☐	☐	☐	☐	☐	☐
**Lesão 5**	☐	☐	☐	☐	☐	☐

14. Você já foi diagnosticado com alguma doença ou lesão no joelho? ☐ Sim ☐ Não

15. Se sim, quais?

☐ Tendinopatia
☐ Ruptura do Ligamento Cruzado Anterior
☐ Ruptura do Ligamento Cruzado Posterior
☐ Ruptura do Ligamento Colateral Lateral
☐ Ruptura do Ligamento Colateral Medial
☐ Lesão de menisco
☐ Osteocondrite dissecante
☐ Síndrome do trato iliotibial
☐ Condropatia patelar
☐ Luxação da patela
☐ Doença de Osgood Schlatter
☐ Outros

16. Se outros, qual diagnóstico? _____________

17. Você já precisou ficar afastado da prática esportiva por alguma lesão no joelho relacionada ao beisebol?

☐ Sim ☐ Não

18. Se já ficou afastado, por quanto tempo?

☐ Não fiquei
☐ Menos de 1 mês
☐ 1 - 3 meses
☐ 3 - 6 meses
☐ Mais de 6 meses

19. Você já fez algum tratamento não cirúrgico por alguma lesão no joelho relacionada ao beisebol?

☐ Sim ☐ Não

20. Se sim, qual?

☐ Nenhum

☐ Medicamentoso

☐ Fisioterapia

☐ Acupuntura

21. Você já fez alguma cirurgia no joelho por alguma lesão do joelho relacionada ao beisebol?

☐ Sim ☐ Não

22. Se sim, qual? ________________

23. Se já fez algum tratamento, você considera que voltou a praticar o esporte após o tratamento no mesmo nível que praticava previamente?

☐ Nunca fiz tratamento ☐ Sim ☐ Não

© 2024. The Author(s).
