# Supplementary material for: Epidemiology of Knee Injuries in Baseball Players from the State of São Paulo
Source: Rev Bras Ortop (Sao Paulo). 2024 Apr 10;59(2):e199–205. doi: 10.1055/s-0044-1785202 (PMC11006523; doi:10.1055/s-0044-1785202)
Supplement: Supplementary file 2 — Supplementary Material, Annex 1 [file 10-1055-s-0044-1785202-s2300208en.pdf]

ANNEX 1

QUESTIONNAIRE – Knee pain in baseball players

Demographic data

1. Age:
2. Gender: ☐ Male ☐ Female
3. Race / Color / Ethnicity: ☐ Yellow ☐ White ☐ Brown ☐ Black ☐ Native ☐ Other:
4. Education:

☐ Incomplete elementary education ☐ Complete elementary education

☐ Incomplete high school education ☐ Complete high school education

☐ Incomplete college education ☐ Complete college education

Activity

5. In which position do you play?

☐ Pitcher ☐ Second base ☐ Left Fielder

☐ Catcher ☐ Shortstop ☐ Center Fielder

☐ First base ☐ Third base ☐ Right Fielder

6. How many hours a week do you play baseball?

☐ Up to 5 hours

☐ From 5 to 10 hours

☐ From 10 to 15 hours

☐ From 15 to 20 hours

☐ More than 20 hours

7. How long have you been playing baseball?

☐ Less than 12 months ☐ Over 12 months

8. Do you practice other sports? Which ones? ☐ No ☐ Yes: ______________________

9. How much time do you play sports other than baseball per week?

☐ Up to 5 hours

☐ From 5 to 10 hours

☐ From 10 to 15 hours

☐ From 15 to 20 hours

☐ More than 20 hours

10. How long have you been playing these other sports?

☐ ☐ Less than 12 months ☐ Over 12 months

Symptoms and Diagnoses

11. Do you have any complaints or symptoms involving your knee?

☐ No ☐ Pain in the front of the knee

 © 2024. The Author(s).

☐ Pain in the back of the knee
☐ Pain on the sides of the knee
☐ Edema (swelling)
☐ Instability
☐ Locking
☐ Crepitus (popping)

12. How many times have you injured your knee (through trauma)? ________________

13. What was the mechanism of injury to the knee?

	No contact	Contact with the ground	Contact with the ball	Contact with another player	Contact with object	Other
**Injury 1**	☐	☐	☐	☐	☐	☐
**Injury 2**	☐	☐	☐	☐	☐	☐
**Injury 3**	☐	☐	☐	☐	☐	☐
**Injury 4**	☐	☐	☐	☐	☐	☐
**Injury 5**	☐	☐	☐	☐	☐	☐

14. Have you ever been diagnosed with any knee condition or injury? ☐ Yes ☐ No

15. If so, which ones?

☐ Tendinopathy
☐ Anterior cruciate ligament rupture
☐ Posterior cruciate ligament rupture
☐ Lateral collateral ligament rupture
☐ Medial collateral ligament rupture
☐ Meniscus injury
☐ Osteochondritis dissecans
☐ Iliotibial band syndrome
☐ Patellar chondropathy
☐ Patellar dislocation
☐ Osgood-Schlatter disease
☐ Others

16. If others, what was the diagnosis? _____________

17. Have you ever had to take time off from playing sports due to a baseball-related knee injury?

☐ Yes ☐ No

18. If you have taken time off, for how long?

☐ None
☐ Less than a month
☐ 1 to 3 months
☐ 3 to 6 months
☐ Over 6 months

 © 2024. The Author(s).

19. Have you ever had non-surgical treatment for a baseball-related knee injury?

☐ Yes ☐ No

20 If yes, which one? ☐ None

☐ Medication

☐ Physical therapy

☐ Acupuncture

21. Have you ever had knee surgery for a baseball-related knee injury?

☐ Yes ☐ No

22. If yes, which one? ________________

23. If you have undergone treatment, do you consider that you returned to baseball practice after treatment at the same level as before?

☐ I have never had treatment ☐ Yes ☐ No
