# Supplementary material for: Epidemiology of Knee Injuries in Baseball Players from the State of São Paulo
Source: Rev Bras Ortop (Sao Paulo). 2024 Apr 10;59(2):e199–205. doi: 10.1055/s-0044-1785202 (PMC11006523; doi:10.1055/s-0044-1785202)
Supplement: Related texts

# Research article: Epidemiologia das lesões do joelho em atletas de beisebol do estado de São Paulo

**Keywords:** beisebol, joelho, traumatismos do joelho, lesões do menisco tibial, tendinopatia

## Abstract

**Objetivo** Identificar as principais queixas e lesões de joelho associadas ao beisebol, e sua prevalência em atletas de beisebol no estado de São Paulo.

**Métodos** Estudo epidemiológico desenvolvido por meio da análise de dados obtidos por um questionário *online* , entre os anos de 2019 e 2022, distribuído entre atletas de beisebol do estado de São Paulo.

**Resultados** Noventa e oito atletas participaram do estudo, com média de 24,3 anos de idade, sendo que 85,72% eram homens. As etnias mais prevalentes foram os amarelos (50%) e brancos (42,86%), e a maioria dos atletas possuía ensino superior incompleto ou completo (75,5%). Um total de 88,77% treinava há mais de 1 ano e 40,82% atuavam em mais de uma posição. Mais da metade praticava simultaneamente outro esporte. Um total de 66,32% dos atletas apresentava queixas ou sintomas no joelho e 37,75% já haviam sofrido alguma lesão no joelho associada à prática do beisebol através de diversos mecanismos (contato com solo, contato com outro jogador, sem contato). Um total de 59,45% dos atletas precisou ser afastado da prática devido às queixas, sintomas ou lesões apresentadas.

**Conclusão** Dos atletas entrevistados, 66,32% apresentaram alguma queixa no joelho e 37,75% já tiveram alguma lesão diagnosticada nessa articulação, sendo as mais prevalentes as lesões meniscais e as ligamentares. A taxa de lesões foi maior no primeiro ano de prática.

## Introdução

As lesões musculoesqueléticas são comuns na prática esportiva e correspondem a cerca de 80% das lesões no esporte. 1 Lesões articulares, principalmente no joelho, têm aumentado significativamente em decorrência do crescimento do número de pessoas que praticam atividade física tanto profissional quanto recreativamente. Além disso, essas práticas têm iniciado numa faixa etária cada vez mais precoce, com uma solicitação de treinamentos e níveis de competitividade cada vez maiores, 2 3 algumas vezes desprovidas de supervisão técnica apropriada.

Existe uma concentração dos estudos sobre lesões em atletas de beisebol direcionadas aos membros superiores, visto que essas correspondem a cerca de 45% das lesões ocorridas tanto durante o treino quanto durante o jogo. Oitenta por cento dos artigos publicados baseados na *Major League Baseball* são relativos aos membros superiores, enquanto 6,6% aos inferiores. 4 Entretanto, as lesões dos membros inferiores também estão presentes nesse esporte, correspondendo à cerca de 33% do total de lesões nessa modalidade. 5 6 7 Apesar disso, na literatura não são encontrados tantos estudos direcionados ao tema.

Em vista da escassez de literatura sobre as lesões do joelho nessa modalidade e a ausência de dados epidemiológicos nacionais precisos que nos permitam compreender o panorama delas, este trabalho visa identificar as principais queixas e lesões associadas ao beisebol, e sua prevalência em nosso meio, e assim iniciar uma discussão sobre a necessidade de implementar medidas preventivas na prática segura do beisebol.

## Métodos

Entre maio de 2019 e maio de 2022, após aprovação do Comitê de Ética e Pesquisa (CAAE: 17721819.0.0000.5479), os pesquisadores atrelados ao Grupo do Joelho do Departamento de Ortopedia e Traumatologia do mesmo hospital solicitaram a diversos clubes de beisebol do estado de São Paulo e equipes universitárias que distribuíssem entre seus atletas um questionário *online,* via *Google Forms* , que incluiu uma versão digital do termo de consentimento livre e esclarecido, seguido de questões específicas para identificação demográfica, epidemiológica e lesões dos joelhos dos mesmos. O questionário foi desenvolvido e avaliado internamente pelos membros do grupo ao longo de três rodadas de revisão, e a versão final foi diretamente aplicada aos atletas de modo a coletar dados relativos à prática de beisebol.

Foram convidados a responder o questionário ( Material Suplementar, Anexo 1 ), voluntariamente e por autopreenchimento, os atletas amadores associados à Liga Esportiva das Atléticas de Medicina do Estado de São Paulo (LEAMESP) e dos clubes: Anhanguera, Atibaia, Gecebs, Nippon Blue Jays, São Paulo Gigante, Los Tomateros, Underdogs e UniSant'Anna. O questionário foi difundido pelo aplicativo *WhatsApp* pelo representante de cada instituição, de modo a atingir 195 atletas no total.

Os critérios de inclusão foram atletas amadores de beisebol do estado de São Paulo, independentemente da idade, sexo ou etnia, que praticam a modalidade ao menos uma vez por semana, em diversos níveis de desempenho e afiliados às equipes supracitadas, sendo incluídos os que responderam ao questionário e termo de consentimento de forma completa e correta. Os critérios de exclusão previamente definidos foram: atletas que sofreram lesões musculoesqueléticas decorrente da prática de outro esporte, que não preencheram o questionário adequadamente e para-atletas, aqueles que apresentam alguma deficiência física, visual ou intelectual.

## Descrição do questionário

O questionário, elaborado pelos autores, é composto por 23 questões: 4 sobre dados demográficos, 6 referentes à prática esportiva e 13 relacionadas a sintomas, diagnósticos e tratamentos específicos de lesões do joelho, com base na necessidade de identificar as questões epidemiológicas envolvendo essa população.

Na seção de dados demográficos foram solicitadas e obtidas informações como sexo, idade, etnia e escolaridade. Na seção sobre a prática esportiva do beisebol foram coletados dados como tempo de prática do esporte superior ou inferior a um ano, tempo dedicado ao treino em horas semanais, e posição em que o atleta joga – arremessador, apanhador, *infielders* (primeira base, segunda base, terceira base e interbases) e *outfielders* (campista esquerdo, campista central e campista direito). Além destes, coletaram-se dados sobre a prática de outros esportes e sobre o tempo de treino em horas semanais e tempo de prática deste, superior ou inferior a um ano.

O questionário também continha perguntas quanto à sintomas, diagnósticos e tratamentos específicos do joelho, como: presença de queixa clínica no joelho como dor e sua topografia, episódio de instabilidade, edema, travamento e crepitação; presença de diagnósticos específicos de lesões ao longo da prática do esporte; quais foram esses diagnósticos, considerando que um atleta poderia ter recebido mais de um; mecanismos envolvidos nas lesões (sem contato, contato com a bola, com o solo, com objeto, com outro jogador e outros); necessidade de afastamento por queixa clínica ou lesão; tempo do afastamento em meses; submissão a tratamentos conservadores; quais intervenções dessa natureza; submissão a tratamentos cirúrgicos e quais foram eles.

## Análise dos dados

Quanto aos dados demográficos, a idade foi exposta por meio da média e desvio padrão e o sexo, etnia e escolaridade foram expostos em números absolutos e relativos. Também foram expostas dessa maneira as informações obtidas referentes à prática do esporte e aos sintomas, diagnósticos e tratamentos específicos do joelho.

Os demais dados foram tabelados e expostos conforme explicado previamente. Idade, tempo de prática do esporte em anos, horas de treino semanais, posição em que joga e prática de outro esporte foram analisados de forma a ressaltar a possibilidade de associações entre a presença de queixas ou lesões com algumas variáveis do estudo.

Para esta análise secundária, os participantes foram estratificados de formas distintas e foi observada a distribuição de queixas e lesões em cada subgrupo. Para a idade, tomou-se como base a mediana dos valores dessa variável, para a prática do esporte em anos, foram estratificados em um grupo que praticava a menos de um ano e outro igual ou superior a um ano baseando-se em outros trabalhos que evidenciam diferenças nas taxas de lesões para atletas subdivididos dessa maneira, 8 9 para as horas de treino semanais considerou-se as cinco categorias presentes no questionário, quanto à atuação no esporte, foi considerado o desempenho de uma ou mais posições no beisebol e, quanto à prática de outros esportes, foram subdividos em um grupo que pratica somente beisebol e outro que pratica outras modalidades esportivas.

## Resultados

Noventa e oito de 195 atletas preencheram o questionário voluntariamente, correspondendo a uma taxa de resposta de 50,25% e apresentaram média de idade de 24,3 anos ± 7,1, sendo 84 homens (85,72%) e 14 mulheres (14,28%). Não houve exclusão de nenhum atleta após o preenchimento do questionário. Foram encontradas quatro etnias distintas, 49 (50%) amarelos, 42 (42,86%) brancos, 6 (6,12%) pardos, e 1 (1,02%) preto. Entre os atletas, 75,5% declaravam possuir ensino superior incompleto ou completo ( Tabela 1 ).

**Tabela 1:** Dados demográficos em média, desvio padrão e mediana ou números absolutos e relativos dos participantes

**Idade**	**Média (DP)**	**Mediana**			
	24,33 (±7,1)	23			
Sexo	Masculino	Feminino			
	84 (85,72%)	14 (14,28%)			
Raça	Amarela	Branca	Parda	Preta	
	49 (50%)	42 (42,86%)	6 (6,12%)	1 (1,02%)	
Escolaridade	Ensino fundamental incompleto	Ensino médio incompleto	Ensino médio completo	Ensino superior incompleto	Ensino Superior Completo
	5 (5,1%)	4 (4,08%)	15 (15,4%)	52 (53,06%)	22 (22,44%)

Abreviações: DP, desvio padrão; %, número relativo expresso em percentual.

Em relação à prática específica do beisebol, 87 (88,77%) treinam a modalidade por tempo superior a 1 ano, 24 (24,48%) treinam mais que 10 horas semanais, 40 (40,82%) atuam em pelo menos 2 posições; a maior parte dos atletas joga como *infielder* ou *outfielder* (51,02% e 47,95%, respectivamente), e 50 (51,02%) atletas declararam estar envolvidos na prática de outros esportes ( Tabela 2 ).

**Tabela 2:** Dados referentes à prática esportiva em números absolutos e relativos

**Tempo de prática (N = 98)**	**Inferior a 1 ano**	**Superior a 1 ano**			
	11 (11,23%)	87 (88,77%)			
Tempo de treino (h/sem) (N = 98)	Até 5 h	5–10 h	10–15 h	15–20 h	Mais de 20 h
	36 (36,73%)	38 (38,77%)	17 (17,34%)	6 (6,12%)	1 (1,02%)
Posições*	Arremessador	Apanhador	*Infielder*	*Outfielder*	
	30 (30,61%)	18 (18,36%)	50 (51,02%)	47 (47,95%)	
Atuação (N = 98)	Única	Mais de uma			
	58 (59,18%)	40 (40,82%)			
Outros esportes (N = 98)	Sim	Não			
	50 (51,02%)	48 (48,97%)			
Tempo de treino (h/sem) da outra modalidade (N = 50)	Até 5 h	5–10 h	10–15 h	15–20h	Mais de 20h
	21 (42%)	18 (36%)	7 (14%)	3 (6%)	1 (2%)
Tempo de prática da outra modalidade (N = 50)	Inferior a 1 ano	Superior a 1 ano			
	4 (8%)	46 (92%)			

Abreviações: N, número de participantes no grupo; %, número relativo expresso em percentual; *os participantes podem atuar em mais de uma posição.

Sessenta e cinco atletas (66,32%) se queixaram de algum sintoma no joelho e 37 (37,75%) já foram diagnosticados com alguma lesão no joelho associada à prática de beisebol, dos quais, 14 (37,83%) apresentaram diagnóstico de lesões ligamentares, 14 (37,83%) lesões meniscais diversas e 10 (27,02%) tendinopatias. Vale ressaltar que 16 (43,24%) dos atletas já diagnosticados declararam a ocorrência de mais de uma lesão. Quanto aos mecanismos de trauma, 10 (45,45%) atletas se lesionaram com contato com o solo, 8 (36,36%) sem contato e 4 (18,18%) com contato com outro jogador. Vinte e dois (59,45%) referiram necessidade de afastamento ao longo da prática do esporte por queixa clínica ou lesão, sendo que destes, 5 (22,72%) foram submetidos a tratamento cirúrgico ( Tabela 3 ).

**Tabela 3:** Dados referentes a sintomas, diagnósticos e tratamentos específicos do joelho em números absolutos e relativos

**Sintomas**	**Nenhum**	**Dor anterior**	**Dor posterior**	**Dor lateral**	**Edema**	**Falseio**	**Travamento**	**Crepitação**
	42 (33,9%)	49 (39,5%)	11 (8,9%)	36 (29%)	9 (7,3%)	15 (12,10%)	3 (2,4%)	44 (35,5%)
**Lesão diagnosticada**	Sim	Não						
	37 (37,75%)	61 (62,24%)						
**Lesões (N = 37)**	Tendinopatia	Ruptura do LCA	Ruptura do LCP	Ruptura do LCL	Ruptura do LCM	Multiligamentar	Menisco	Osteocondrite dissecante
	10 (27,02%)	10 (27,02%)	3 (8,1%)	2 (5,4%)	1 (2,7%)	2 (5,4%)	14 (37,83%)	0 (0%)
	STIT	Condropatia Patelar	Luxação patelar	Osgood Schlatter	Cisto de Baker			
	0 (0%)	10 (27,02%)	7 (18,91%)	3 (8,1%)	1 (2,7%)			
**Mecanismos de lesão do joelho (N = 22)**	Contato com bola	Contato com solo	Contato com objeto	Contato com outro jogador	Sem contato			
	0 (0%)	10 (45,45%)	0 (0%)	4 (18,18%)	8 (36,36%)			
**Necessidade de afastamento decorrente de lesão do joelho (N = 37)**	Sim	Não						
	22 (59,45%)	15 (40,54%)						
**Tempo de afastamento (N = 22)**	Até 1 mês	1–3 meses	3–6 meses	Mais de 6 meses				
	7 (31,81%)	8 (36,36%)	4 (18,18%)	3 (13,63%)				
**Tratamento não cirúrgico decorrente de lesão do joelho (N = 37)**	Sim	Não						
	21 (56,75%)	16 (43,25%)						
**Tratamento cirúrgico decorrente de lesão do joelho (N = 37)**	Sim	Não						
	5 (13,51%)	32 (86,49%)						

Legenda: LCA, ligamento cruzado anterior; LCL, ligamento colateral lateral; LCM, ligamento colateral medial; LCP, ligamento cruzado posterior; N, número de participantes no grupo; %, número relativo expresso em percentual; STIT, síndrome do trato iliotibial.

Para a análise secundária, os atletas foram inicialmente subdivididos em 2 grupos com base na mediana da idade: 23 anos ( Tabela 1 ). Notou-se que, dos atletas com idade superior a 23 anos, 71,11% apresentavam queixas clínicas referentes aos joelhos e 35,55% já foram diagnosticados com alguma lesão nessa articulação.

Foi observado que 72,72% (8 entre 11) dos atletas que praticam beisebol há menos de 1 ano apresentaram queixas clínicas no joelho e 45,45% (5 dos 11), já receberam algum diagnóstico de lesão no joelho. Dentre os atletas que praticam há mais de um ano, 65,51% apresentavam queixas (57 de 87) e 36,78% lesões (32 de 87) ( Tabela 4 ).

**Tabela 4:** Presença de sintomas e lesões diagnosticadas em relação ao tempo de prática

	Todos (N = 98)	Mais de um ano (N = 87)	Menos de um ano (N = 11)
Sintomas	65 (66,32%)	57 (65,51%)	8 (72,72%)
Lesões diagnosticadas	37 (37,75%)	32 (36,78%)	5 (45,45%)

Abreviações: N, número de participantes no grupo; %, número relativo expresso em percentual.

Entendendo a limitação da distribuição de pessoas por subgrupo na condição de queixas e lesões por horas de treino semanais, destaca-se que 66,66% dos 36 atletas que treinam até 5 horas semanais apresentam queixas e 54,16% delas, lesões diagnosticadas ( Tabela 5 ). Além disso, dos 40 indivíduos que treinavam em mais de uma posição, 72,5% e 42,5%, relataram apresentar sintomas ou lesões no joelho, respectivamente; dos 50 que praticavam outros esportes, 66% apresentavam queixas e 34% relatavam lesões e dos 48 que praticavam apenas o beisebol, 66,66% apresentavam sintomas e 39,58% apresentam lesões diagnosticadas.

**Tabela 5:** Relação do tempo de treino de beisebol (em horas por semana) com a presença de sintomas e lesões diagnosticadas em números absolutos e relativos

Horas/semana	Sintomas	Lesões
Até 5 h (N = 36)	24 (66,66%)	13 (54,16%)
5–10 h (N = 38)	25 (65,78%)	12 (31,57%)
10–15 h (N = 17)	12 (70,58%)	6 (35,29%)
15–20 h (N = 6)	3 (50%)	3 (50%)
Mais de 20 h (N = 1)	1 (100%)	1 (100%)

Abreviação: N, número de participantes no grupo; %, número relativo expresso em percentual.

## Discussão

Esse estudo visa expor os dados epidemiológicos sobre sintomas e lesões musculoesqueléticas do joelho em atletas amadores de beisebol do estado de São Paulo, com o intuito de auxiliar o cuidado com a saúde do atleta, contribuindo com informações para criação de medidas preventivas e regulamentações da prática do beisebol. É de conhecimento dos autores que este seja o primeiro estudo a avaliar a epidemiologia das lesões do joelho nos atletas desse esporte em contexto nacional.

Segundo a Lei 9.615 de 1998 do Governo Federal, que dispõe sobre normas gerais da prática esportiva, são definidos como atletas profissionais aqueles que apresentam remuneração definida em contrato formal de trabalho. Dessa maneira, todos os atletas deste estudo enquadram-se como não-profissionais (amadores).

Sabe-se que nas principais ligas americanas ( *Major League Baseball* e *Minor League Baseball* ) as lesões do ombro correspondem a 14,7% de todas as lesões, enquanto o joelho é acometido em 6,5% do total. 6 Esses atletas, apesar de utilizarem predominantemente os membros superiores para o movimento de arremesso, também exercem esforços intensos sobre os membros inferiores para a corrida em linha reta, mudança de direção súbita durante a corrida, saltos e, dependendo da posição que o atletas exerça, permanece longos períodos agachado, mantendo ou realizando o movimento de flexão dos joelhos repetidas vezes. 10 Mesmo quanto ao arremesso, a efetividade do movimento está intimamente relacionada à transferência de energia dos membros inferiores para o tronco. 11 Assim, o joelho torna-se uma articulação suscetível a lesões no beisebol, as quais seguem subdiagnosticadas, uma vez que é escassa a quantidade de trabalhos que se destinam a averiguá-las.

Em estudo conduzido no Japão, 12 durante uma análise de jovens atletas de beisebol que referiam dor no ombro e/ou cotovelo, observou-se que 51,9% deles também se queixavam de dor no joelho. Queixas também apresentadas por 66,32% de todos os atletas participantes do nosso estudo, independente de referirem sintomas nos membros superiores. Um total de 37,5% dos atletas apresentou alguma lesão diagnosticada. Comparados a outro estudo, fundamentado no banco de dados das lesões diagnosticadas nos profissionais de beisebol das ligas norte-americanas, em que as lesões do joelho correspondem a apenas 6,5% do total, 6 nota-se uma heterogeneidade das taxas de lesões em diferentes contextos.

Nota-se na literatura que as principais lesões no joelho apresentadas por atletas dessa modalidade correspondem à contusão (30,5%), 6 lesão ligamentar (17,5%), 6 tendinopatias (16,9%) 6 e lesão meniscal (9,2–33%) 1 6 , que variam em incidência conforme o local do estudo. Luxação e subluxação femoropatelares (1,4%) 6 são diagnósticos citados com menor prevalência. No presente estudo, as principais lesões encontradas foram ligamentares (37,83%), meniscais (37,83%) e tendinopatias (27,02%).

Acreditamos que o caráter amador da prática do beisebol no âmbito regional possa ser responsável pela maior incidência de queixas e de lesões diagnosticadas nos joelhos em nosso estudo, quando comparadas à literatura internacional, que avalia principalmente atletas profissionais. 4 6 Essa discrepância nos faz questionar a necessidade de mais estudos focados nas lesões de joelho nos atletas amadores de beisebol.

Quanto ao tempo de prática, encontramos que atletas que treinam há menos de um ano apresentam maior taxa de lesões diagnosticadas do joelho, o que também é visto em outros esportes, como na corrida de rua, em que estudos realizados desde a década de 80 concluem que atletas menos experientes tendem a sofrer maior número de lesões nos membros inferiores. 13 14 Estudos mais recentes, evidenciam que essa incidência pode ser até duas vezes maior nos atletas considerados iniciantes, durante o primeiro ano de suas práticas. 8 9 Apesar de haver heterogeneidade na amostra, é possível inferir que atletas com menor tempo de prática apresentam maior taxa de lesão, mesmo havendo discrepâncias na caracterização dos tipos de lesões, da sua classificação quanto ao nível de rendimento e da dificuldade encontrada para avaliar o momento em que elas ocorrem. Assim, é necessário realizar novos trabalhos nessa área e promover cuidados diferenciados para atletas com menor tempo de prática.

Em relação ao mecanismo de trauma das lesões diagnosticadas, Dahm et al. 6 apontam que os principais sejam sem contato (43,9%) e contato com solo (18,8%), enquanto na casuística deste estudo, as taxas encontradas foram de 36,36% e 45,45%, para os mecanismos sem contato e com contato com o solo, respectivamente. Randall et al. (2007) apontam que 42% das lesões no beisebol ocorreram sem contato enquanto 45% ocorreram com contato. As principais lesões diagnosticadas no nosso estudo foram as ligamentares e meniscais do joelho, geralmente associadas a traumas torcionais, correspondendo ao mecanismo de trauma sem contato, e corroborando com o cenário descrito.

Existe uma lesão descrita em atletas de beisebol denominada joelho do apanhador ( *catcher's knee* ). 15 É pouco conhecida e, portanto, pode ser subdiagnosticada. Corresponde a uma osteocondrite dissecante da região posterior do côndilo femoral, que é causada pela hiperflexão persistente e repetitiva dessa articulação, mecanismo típico dos movimentos dos atletas dessa posição. Neste estudo, questionou-se especificamente a presença desse diagnóstico na seção de sintomas e lesões do joelho. Os apanhadores não apresentaram um padrão específico de diagnóstico de lesões ou queixas e não tivemos nenhum caso de “joelho do apanhador” entre os atletas da nossa casuística.

Os participantes deste estudo apresentaram altas taxas de sintomas e/ou lesões diagnosticadas nos joelhos, assemelhando-se inclusive às encontradas em outros estudos, na avaliação dos membros superiores de atletas de alto rendimento. 4 5 6 Atualmente, até onde é de conhecimento dos autores, no Brasil não existe diretriz que oriente a prática desse esporte ou o uso de equipamentos de modo a prevenir lesões do joelho, o que pode estar relacionado à diferença nos dados observados quando se comparam atletas amadores com profissionais ou à carência de estudos voltados à epidemiologia dessas lesões.

Este estudo apresenta algumas limitações, como a amostra pequena e heterogênea, apesar da ampla distribuição do questionário nas ligas; dificuldade em interpretar e associar a presença de queixas e lesões a variáveis como a posição em que o atleta joga devido à prática em diferentes posições pelo mesmo indivíduo. Apesar deste estudo apontar percepções interessantes sobre o tema lesões no joelho e a prática amadora de beisebol no Brasil, acredita-se que um novo estudo com cálculo de amostra e provavelmente maior casuística possa ser necessário para que sejam obtidas relações e análises mais precisas.

## Conclusão

Dos atletas entrevistados, 66,32% apresentavam alguma queixa no joelho e 37,75% já haviam apresentado alguma lesão diagnosticada nessa articulação, sendo as mais prevalentes as lesões meniscais e as ligamentares. A taxa de lesões foi maior no primeiro ano de prática do esporte.
